# Isolated abdominal wall recurrence of pancreatic ductal adenocarcinoma: a rare case report

**DOI:** 10.1093/jscr/rjae418

**Published:** 2024-06-21

**Authors:** Tegan Lun, Pranavan Palamuthusingam, Nicholas O’Rourke

**Affiliations:** Department of Surgery, Townsville University Hospital, Townsville, 4814, QLD, Australia; Department of Surgery, Royal Brisbane and Women’s Hospital, Brisbane, 4006, QLD, Australia; Department of Surgery, Wesley Hospital, Brisbane 4066, QLD, Australia; Department of Surgery, Royal Brisbane and Women’s Hospital, Brisbane, 4006, QLD, Australia; Department of Surgery, Wesley Hospital, Brisbane 4066, QLD, Australia

**Keywords:** pancreatic ductal adenocarcinoma, abdominal wall, isolated recurrence, resection, case report

## Abstract

A 76-year-old woman was investigated for epigastric pain on a background of a laparoscopic distal pancreatectomy and splenectomy for pancreatic ductal adenocarcinoma 4 years prior. Imaging revealed an isolated 32 mm fluorodeoxyglucose avid lesion contacting both the anterior abdominal wall and greater curvature of the stomach. Immunohistochemistry and fine needle biopsy confirmed a phenotype consistent with metastatic pancreatic adenocarcinoma. Laparoscopic excision of the mass and partial gastrectomy for clearance of margins was performed. Histopathology demonstrated a poorly differentiated pancreatic ductal adenocarcinoma, and the patient received adjuvant gemcitabine/capecitabine following an uncomplicated postoperative course. This article presents a rare case of isolated abdominal wall recurrence of pancreatic ductal adenocarcinoma, which was successfully treated with surgical resection and adjuvant chemotherapy.

## Introduction

Despite advances in diagnostic methods, surgical techniques, and adjuvant therapies, the prognosis of pancreatic ductal adenocarcinoma (PDAC) remains poor. Even with curative resection, 5-year survival remains less than 20%, and recurrence rates are as high as 80% [1, 2]. Surgery for recurrent PDAC has historically been used as a palliative treatment; however, studies have shown that resection of isolated local recurrence (ILR) is safe and can improve survival in selected patients [3–6]. In contrast, there are few studies evaluating the benefit in isolated distant recurrence, which occurs in up to 40% of patients [6, 7]. We present a rare case of isolated abdominal wall recurrence, which was successfully treated with surgical resection and adjuvant chemotherapy.

## Case report

A 76-year-old woman was referred to our practice with 3 months of intermittent epigastric pain. Her past medical history was significant for a 24 mm PDAC 4 years prior, which was treated with six cycles of neoadjuvant FOLFIRINOX, laparoscopic distal pancreatectomy and splenectomy, and six cycles of adjuvant capecitabine/gemcitabine. The final histology showed R0 resection, poor differentiation, and vascular invasion without lymph node metastasis (0/11)**.** Annual computed tomography (CT) had showed no recurrence. On review, examination was unremarkable. Laboratory investigations showed an elevated white cell count of 12.8 ×10^9^/L (3.5–10.0 ×10^9^/L), but no elevation of carcinoembryonic antigen (<5.0 μg/L), carbohydrate antigen 19-9 (<34 U/ml), or carbohydrate antigen 125 (<35 U/ml).

**Figure 1:**
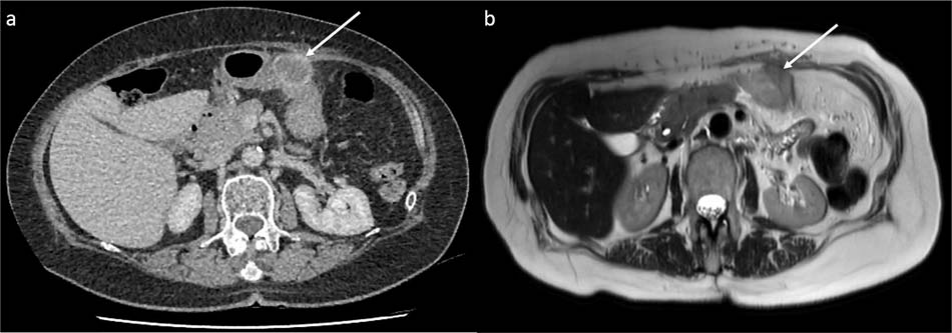
CT and MRI of the lesion (arrows). (a) CT showed a 32 mm cystic enhancing lesion between the anterior abdominal wall and mid greater curvature of the stomach. (b) The lesion appeared hyperintense on T2 imaging and predominantly isointense on T1 imaging, without significant internal debris.

CT showed a 32 mm cystic enhancing lesion contacting both the anterior abdominal wall and greater curvature of the stomach (Fig. 1a). Magnetic resonance imaging (MRI) suggested that the mass was more likely solid than cystic. It was hyperintense on T2 imaging, predominantly isointense on T1 imaging, and showed peripheral enhancement in the early arterial phase without restricted diffusion or significant internal debris (Fig. 1b). A fluorodeoxyglucose (FDG) positron emission tomography scan showed that the mass was lobulated with heterogenous peripheral FDG uptake. CT head and chest were normal. Gastroscopy and mucosal biopsy showed a submucosal mass along the greater curvature of the stomach without mucosal breach, suggesting origin from the abdominal wall. Endoscopic ultrasound was performed with fine needle biopsy. Histopathology showed poorly differentiated malignancy, and immunohistochemistry confirmed a phenotype consistent with metastatic PDAC.

**Figure 2:**
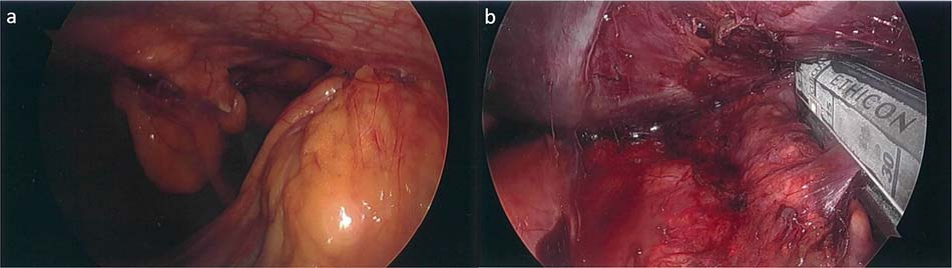
Laparoscopic images. (a) Tumor with a broad base on the anterior abdominal wall and apex adherent to the greater curvature of the stomach inferiorly. (b) Partial gastrectomy for clearance of margins, following tumor excision.

**Figure 3:**
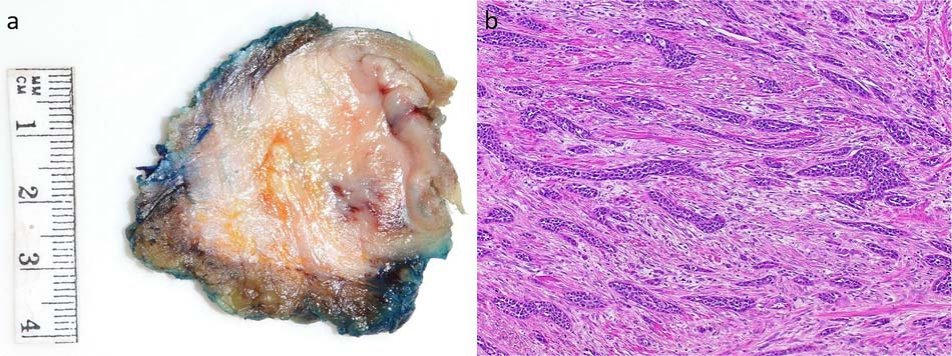
Macroscopic and microscopic appearance of the tumor. (a) A 33 × 30 mm mural tumor. (b) Hematoxylin and oesin (HE) stain at 40× magnification showing invasive ductal adenocarcinoma with prominent desmoplastic stroma.

Subsequently, the patient underwent a laparoscopic excision of the mass and partial gastrectomy for clearance of margins. During laparoscopy, it was found that the tumor had a broad base on the anterior abdominal wall overlying suture lines from the original surgery, with a small area at its apex adherent to the greater curvature of the stomach (Fig. 2a and b). Histopathology showed a 33 mm poorly differentiated PDAC with peritoneal surface involvement, but clear margins (Fig. 3a and b). The postoperative course was uncomplicated, and followed by six cycles of adjuvant gemcitabine (1530 mg on Days 1, 8, and 15)/capecitabine (2150 mg on Day 1). The patient had a progression free survival of 15 months, before declining further chemotherapy for new liver metastases. At 24 months postoperatively, the patient remains well and is receiving symptomatic management by palliative care.

## Discussion

Despite recurrence rates of up to 80% after R0 resection, there are no standardized guidelines for the management of recurrent PDAC, and many patients are treated using chemotherapy alone with palliative intent [2]. Although numerous chemotherapy regimens have demonstrated survival benefit, median survival after recurrence remains as low as 9 months [7]. With considerable rates of isolated recurrence, there has been increasing research into the role of surgical resection for different sites.

A number of non-randomized retrospective studies have shown that resection of ILR is safe and can improve survival in selected patients [3–6]. In 2021, Serafini *et al*. [3] conducted a systematic review and meta-analysis on the outcome of resection for ILR in 431 patients, with or without chemotherapy. The authors reported a perioperative mortality rate of 1.1%, and median overall survival benefit of 28.7 months.

In contrast, there are currently few studies evaluating the benefit of resection for isolated distant recurrence. In 2019, Kim *et al.* [6] published a non-randomized retrospective study including 197 patients with isolated locoregional or distant recurrence, which found that the patients who underwent resection demonstrated a longer median survival (23.5 months vs 12 months) in the absence of major postoperative complications. Similarly to ILR, re-resection for isolated distant recurrence has also been associated with better survival after recurrence rates than chemotherapy ± radiotherapy at 1 year (62% vs 40%) [4]. In particular, patients with isolated lung metastases have been found more likely to benefit than recurrence at other sites [2, 8]. Notably, isolated recurrence of the abdominal wall is quite rare. To the best of our knowledge, there are only three reports of such recurrence published at the time of writing. All three patients underwent re-resection and remained well at the last follow-up of 16 months, 43 months, and 72 months, despite no additional systemic therapy [9–11].

The exact subgroup to benefit from surgery is yet to be defined; however, positive prognostic factors for survival after recurrence are well established. These include: age < 65-years-old, body mass index > 20 kg/m^2^, low American Society of Anesthesiologists Physical Status, non-elevated CA 19.9 (<37 U/ml), normal serum albumin level, tumor size < 30 mm, well-differentiated primary tumor with resection margins of >10 mm and node-negativity, disease free interval > 10 months, and chemotherapy for recurrence [4–6]. Our patient demonstrated most of these factors, with the exception of her age and tumor size. Considering her paucity of comorbidities and significant disease free interval of 46 months, she was offered surgical resection.

## Conclusion

The current literature indicates that resection for isolated recurrence offers survival benefit in select patients. However, given that this literature is subject to selection bias, no definitive selection criteria have been established, and the most appropriate treatment modalities for distant recurrence remain unclear, all cases should continue to undergo discussion at a multidisciplinary level. Our case aligns with the existing reports of isolated abdominal wall recurrence, which indicate that resection can be performed safely with the potential to achieve long-term survival.
